# Age- and sex-related differences in landmark recall following a virtual spatial navigation task

**DOI:** 10.3389/fnagi.2025.1602945

**Published:** 2025-08-13

**Authors:** Alexis N. Chargo, Cheryl L. Dahle, Naftali Raz, Ana M. Daugherty

**Affiliations:** ^1^Institute of Gerontology, Wayne State University, Detroit, MI, United States; ^2^Department of Psychology, Wayne State University, Detroit, MI, United States; ^3^Department of Psychology, Stony Brook University, Stony Brook, NY, United States; ^4^Max Planck Institute for Human Development, Berlin, Germany

**Keywords:** cognitive map, landmark recall, navigation cues, spatial navigation, wayfinding

## Abstract

**Introduction:**

Wayfinding is a cognitive ability that supports accurate spatial navigation and declines in this ability adversely affect independent living in older age. The cognitive map represents environmental details, such as landmark cues, relative to the goal location. Distal cues appear to be less effective than proximal ones in precisely locating the goal. Age-related declines in spatial precision may result in fewer accurate landmark-place details or hinder the differential use of cue types.

**Methods:**

This study examined spatial navigation abilities using a virtual adaptation of the Morris Water Maze in a community lifespan sample of 169 adults (aged 18–78 years). Following 25 learning trials with a fixed, hidden platform, spatial precision of recalling the platform location was tested with map reproduction tasks that manipulated the environmental presentation of cues.

**Results:**

Age-related differences varied by sex; middle-aged and older women were less precise in recalling platform location compared to men, but only when provided with all distal and proximal cues. This effect was partially related to the recall accuracy of landmark-place associations: middle-aged and older women who had recalled fewer details were less precise when provided all landmark cues. By comparison, the association between free recall and spatial precision was weaker in younger women and in middle-aged and older men.

**Discussion:**

These findings suggest differential age- and sex-related variations in the integration of navigation cues in wayfinding.

## 1 Introduction

Wayfinding is a cognitive ability that enables the selection and adaptation of routes to a goal location and is essential for independent living. Community-dwelling older adults report wayfinding difficulty even in familiar environments, and severe declines may be harbingers of dementia (Coughlan et al., 2018; Laczó et al., 2022). The source of these deficits is unclear, as wayfinding relies on complex cognitive processing to orient in the environment, build landmark and route knowledge, plan, monitor, and adapt travel routes in real time (Dalton et al., 2019; Chargo et al., 2024). These details are thought to be stored as a mental representation of the environment, or a cognitive map (Tolman, 1948; O'Keefe and Nadel, 1978). The cognitive map is believed to represent the geometry of the environment, including details of available landmarks, meaningful spatial relationships among them, and their locations, and experienced travel routes (Kimura et al., 2019; Kim and Bock, 2021). The concept of a cognitive map, although not universally accepted, helps to describe the spatial precision and recall of environmental details used during navigation toward a designated goal (Ekstrom and Ranganath, 2018; Farzanfar et al., 2023). Characterizing age-related differences in landmark details included in a cognitive map may provide insights into the nature of wayfinding deficits in older adults.

The cognitive map is continually refined by exploration of the environment and the application of navigation strategies (O'Keefe and Nadel, 1978). Within the cognitive map, different types of cues—proximal and distal, as well as geometry—are encoded to support orientation in the environment and goal-directed navigation. Proximal landmarks are located close to a goal and provide positional information, whereas distal landmarks are positioned farther away and benefit orienting within the environment (Chan et al., 2012; Jabbari et al., 2022). Geometric cues provide additional orienting information by informing about the relative positioning of large surfaces in the environment. In reference to these cues, two types of navigational strategies have been described. Allocentric navigation, which is correlated with the hippocampus and medial temporal lobe cortices, is a landmark-based process that relies on encoding, recall, and recognition of landmark-landmark or landmark-place associations. Indeed, the creation and refinement of a cognitive map are thought to depend mainly on the recruitment of the hippocampal and parahippocampal circuits that include place and grid cells. These specialized processing units have been identified in rodent models, but translational evidence in humans is sparse (Ekstrom et al., 2003; Zhang and Ekstrom, 2013; Hao et al., 2016). In comparison, egocentric navigation—supported by extrahippocampal regions such as the caudate nucleus, frontal and parietal cortices—depends on the position of the individual in the environment in reference to objects or landmarks. These brain regions are vulnerable to decline in typical aging, with the hippocampus exhibiting significant atrophy that accelerates in late life (Raz et al., 2005, 2010; Moffat et al., 2006; Yagi and Galea, 2019). The differential vulnerability of regions is thought to, at least in part, underlie adult age differences in navigation behaviors (Iaria et al., 2003; Ekstrom et al., 2017; Lester et al., 2017; Zhong and Moffat, 2018). Because the cognitive map can represent all cue types, an individual will presumably refine the cognitive map based on their navigational strategy preference while exploring the environment.

Not all cue types appear to be equally effective, contributing to sex- and age-related differences in navigation efficiency and accuracy (Moffat, 2009; Chai and Jacobs, 2010; Nazareth et al., 2019). Men are often recognized as having superior navigation skills, outperforming women in an environment containing primarily distal or geometric cues (Chai and Jacobs, 2010; Nazareth et al., 2019). Notably, men favor route planning strategies that rely on distal and geometric cues, whereas landmark-based route strategies are preferred by women (Boone et al., 2018; Munion et al., 2019). Sex differences in behavior may be associated with circulating hormones and vary according to fluctuations in estrogen and testosterone that occur with advancing age and the onset of menopause (Nowak et al., 2014; Koebele et al., 2017). Independent of sex-related differences, older adults navigate less efficiently when using primarily landmark-based cues during wayfinding (Moffat, 2009; Bécu et al., 2020).

Thus, two sources of age-related decline in precision of spatial navigation may be postulated: a general memory impairment that leads to impoverished cognitive maps and inaccuracy of landmark cues (Thomas, 2007; Meneghetti et al., 2012). In older adults, poor episodic memory (Fouquet et al., 2010; Sauzéon et al., 2016), plausibly due to a general age-related deficit in associative memory (Old and Naveh-Benjamin, 2008; Saverino et al., 2016; Goodroe et al., 2018) may interfere with the amount of detail encoded into the cognitive map. Performance on map reproduction tasks (Rodgers et al., 2012; Muffato et al., 2021) further suggests a loss of precision in recalling the platform location relative to landmark cues following a navigation task. Based on these findings, older adults are believed to encode fewer landmark-to-place details in their cognitive maps and are more prone to error in retrieving these associated cues. Together, these would contribute to the creation of an inefficient and inaccurate cognitive map.

Recent studies have begun to examine the importance of various cue types and their contribution to map recall in older adults (Hébert et al., 2017; Newman and McNamara, 2022). Across experimental paradigms, older adults tend to prioritize associations among landmarks regardless of their relative position in the environment (Iachini et al., 2009; Harris et al., 2012; Lithfous et al., 2014), leading to worse allocentric navigation in older age, and probably accounting for fewer accurate landmark details recalled after the task. Similarly, older individuals tend to disregard distal cues, and focus on proximal cues and object characteristics, thus losing precision in recalling the location of a hidden goal (Moffat and Resnick, 2002; Rodgers et al., 2012; Kimura et al., 2019; McAvan et al., 2021). However, other studies highlight both a preference for distal cues and the persevered ability to use these details to facilitate navigation (Iachini et al., 2009; Harris et al., 2012)—suggesting mixed results across the literature. Although distal and proximal cues are both examples of landmarks commonly used in allocentric navigation, older adults navigate better when provided both cue types compared to having only one (Bates and Wolbers, 2014; Merhav and Wolbers, 2019). This suggests that not only more detailed cognitive maps, but varying cue types, may support greater wayfinding accuracy and efficiency for older adults. Yet, the use of multiple cue types during subsequent recall of the goal location has not been closely studied for age-related differences. Moreover, the majority of studies have examined landmark recall following navigation tasks with few learning trials, and it is unclear if reported age-related differences in recall of these environmental details are due to slowed acquisition during navigation instead of impaired cognitive map recall.

### 1.1 Current study hypotheses

We addressed the reported limitations by examining landmark recall following 25 learning trials in a virtual Morris Water Maze (vMWM)—an established human adaptation (Moffat and Resnick, 2002) of the traditional task widely used in studies of rodent navigation and memory (Morris, 1984). The task requires locating a hidden platform using various landmark cues within the defined environment. The task is administered for a series of learning trials, a process that engages several high-level executive function and memory skills (Daugherty and Raz, 2017). Older adults display slower navigation acquisition in the vMWM. In our previous reports, we have shown that 25 learning trials are sufficient for older adults to reach asymptotic levels of navigation performance (Daugherty et al., 2016). This study design enables the evaluation of individual differences in landmark recall, without the confound of varying rates of learning. Following the task, map reproductions are assessed for spatial precision (measured by platform placement error) and free recall of environmental landmarks in a sample of healthy adults. Based on the reviewed literature the current study tested the following hypotheses:

(1) Age will negatively correlate with spatial precision to indicate reduced accuracy in goal recall relative to landmarks in older adults.
(2) Age differences in spatial precision will depend on cue type, in accord with the literature. At subsequent recall, greater precision will be observed with distal and proximal cues presented together, and when provided with only one type of cue, older adults will have greater precision with proximal cues compared to the distal ones.
(3) Sex differences in spatial precision will depend on cue type. Men will demonstrate greater precision than women when presented with distal and proximal cues together, and when provided only one cue type, greater spatial precision will be observed with distal cues as compared to proximal ones.

## 2 Materials and methods

### 2.1. Participants

The participants (*N* = 169) were recruited from the Metro Detroit area as part of a longitudinal study; portions of the sample were reported previously for tests of navigation efficiency (Daugherty et al., 2016) and the current report is a unique analysis of the map reconstruction tasks. See Table 1 for demographic characteristics of the sample. Participants (age 18–78 years; Mdn = 43.8, IQR = 33.92) had on average an almost full college education (*M* = 15.33 years, SD = 1.91 years) and were all task naïve. The size of this convenience sample of *N* = 169 was sufficient for 80–90% power to detect small effects (Cohen's *d* > 0.1; Critical *F* = 1.54; α = 0.05; Faul et al., 2009) in multivariate regression analysis.

**Table 1:** Sample description.

**Variable**	**Total**	**Female**	**Male**
Sample size	169	108	61
White, *n* (%)	105 (62.1%)	60 (55.6%)	45 (73.8%)
Age (years)	42.42 ± 17.87	41.97 ± 17.35	43.23 ± 18.89
Education (years)	15.33 ± 1.91	15.29 ± 2.00	15.39 ± 1.74
MMSE	28.74 ± 0.99	28.81 ± 0.92	28.62 ± 1.11

Demographic profile of the sample. Sample means and standard deviations are reported (M + SD); Mini-Mental State Examination (MMSE).

Enrollment criteria required participants to be at least 18 years of age, free of a diagnosis of endocrine disease, neurological or psychiatric disorders, and cancer. To screen for probable dementia at enrollment, all participants met the criteria of ≥26 on the Mini-Mental State Examination (Folstein et al., 1975). To screen for depression, a cut-off score of 16 on the Center for Epidemiologic Studies-Depression scale (Radloff, 1977) was used. Participants were right-hand dominant and had attained a score of at least 75% on the Oldfield Dominance Questionnaire (Oldfield, 1971). All participants had normal or mildly impaired hearing and corrected vision of 20/50 or better. The study was approved by Wayne State University's Institutional Review Board (IRB), and all participants provided written informed consent before undergoing testing.

### 2.2. Testing procedures

Spatial navigation performance was assessed using a virtual adaptation of the Morris Water Maze (Daugherty et al., 2016). The virtual Morris Water Maze was administered using a custom-built environment developed with Unreal Tournament software (Unreal Tournament, 2003), and the data were collected between the years of 2005 and 2007. The virtual environment was viewed on a 17-inch computer screen from a first-person perspective. To navigate the virtual environment, participants were instructed to use their dominant (right) hand to control a joystick. Participants traveled at a constant speed during exploration and were able to cease movement at will.

#### 2.2.1. Practice

Before testing, participants completed a practice trial in a virtual pool environment with five visible platforms, labeled A–E hovering in the space above the water level. Participants crossed the visible platforms in alphabetical order.

#### 2.2.2. virtual Morris Water Maze (vMWM)

**Figure 1:**
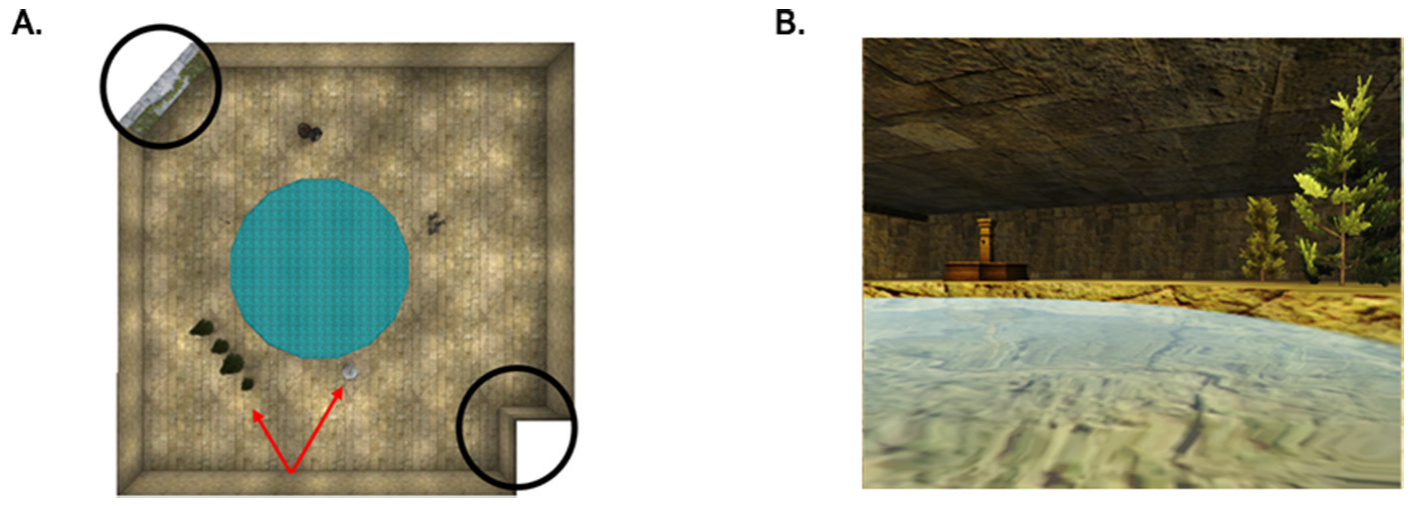
**(A)** Overhead view of the virtual Morris water maze (vMWM) environment. The circular pool (blue) is in the center of a room. Black circles indicate distal cues relative to the position of the platform, and red arrows indicate examples of proximal cues. **(B)** First-person perspective that participants viewed when navigating in the vMWM environment.

The on-screen virtual environment consisted of a circular, water-filled enclosure placed within a square room containing proximal and distal cues relative to a fixed platform hidden beneath the water's surface. See Figure 1 for an overhead depiction and a first-person perspective of the vMWM. Distal cues were considered as the irregularly shaped walls of the room (corner and ivy wall), and proximal cues included five objects (a group of trees, a hanging lamp, a stack of tires, a set of pillars, and a fountain) located around the pool circumference. The platform was centered in one pool quadrant, and five unique starting positions, equidistant from the platform, were assigned to the remaining three quadrants. Each starting position occurred once during a block of 5 trials, resulting in a total of 5 repeated blocks (25 trials in total), with starting positions counterbalanced across blocks and participants. The direction subjects faced at each starting location were randomly set on each trial. Participants were instructed to navigate to the hidden platform as quickly as possible. Learning trials were terminated either by the first intersection with the platform or after 2 min. Navigation efficiency was measured as the total distance traveled from the starting location to the first intersection of the hidden platform, expressed in virtual units (log-transformed to alleviate skew). Refer to Table 2 for the correlations between navigation performance data and map recall tasks. Following the learning trials, a fixed 1-min probe trial and speed control trial were administered. We found a relatively weak and non-significant association between performance on the probe and speed control trial with the outcome measures of spatial precision across all cue types, and therefore these variables were not included for further analysis.

**Table 2:** Bivariate correlations among study variables.

**Variable**	**1**	**2**	**3**	**4**	**5**	**6**
1. Age	–					
2. Map 1: *Free Recall*	−0.15	–				
3. Map 2: *Distal Cues*	0.02	−0.18^*^	–			
4. Map 3: *Proximal Cues*	0.31^**^	−0.27^**^	0.17^*^	–		
5. Map 4: *All Cues*	0.28^**^	−0.28^*^	0.25^**^	0.66^**^	–	
6. Average distance	0.46^**^	−0.35^**^	0.12	0.49^**^	0.45^**^	–

Pearson r correlations describing the relation between map outcome variables (free recall total correct, and map placement error across cue types), age and log-transformed average distance traveled across navigation trials. Map 1: Number of details correctly remembered during free recall task; Map 2: Platform placement error when only provided distal cues; Map 3: Platform placement error when only provided proximal cues; Map 4: Platform placement error when provided all cues.

^*^p < 0.05, ^**^p < 0.01.

#### 2.2.3. Map recall tasks

Following 25 learning trials in the vMWM, participants completed four map tasks. First, participants drew an overhead view of the virtual environment on a blank sheet of paper, including as many details as possible, and marking the location of the hidden platform. Participants' drawings were divided into quadrants, centering the platform within one quadrant. Free recall was scored based on the number of cues included in the map reproduction (i.e., presence of outer walls, objects located around pool perimeter) and their correct (object-quadrant) placement relative to the platform. The maximum possible score was 17. Following free recall, participants were tested for platform location by cued recall. Participants were provided printed, overhead views of the environment and instructed to mark the location of the platform with an “X” (Moffat and Resnick, 2002; Rodgers et al., 2012). Three types of modified maps were presented in that fixed order: those with only distal cues, only proximal cues, and proximal and distal cues together. A scoring transparency, marked with the actual location of the platform, was aligned over the participant's map. Placement error (or spatial precision) was measured as distance (in millimeters, mm) from the participant's marked “X” to the proper location of the platform center (Rodgers et al., 2012).

#### 2.2.4. Control variables

Participants completed a 7-point Likert rating scale (1 = never, 7 = almost every day) to assess prior computer experience and experience playing video games that contained a three-dimensional (3D) environment. Following the completion of the vMWM and map recall, participants reported 17 potential symptoms related to motion sickness on a 10-point Likert scale (0 = not at all, 9 = severely), which were summed.

### 2.3. Statistical analyses

Descriptive statistics, including bivariate correlations of map recall task scores with average distance traveled and control variables, were computed. Hypotheses were tested using a 3 (Cue Type, Within-subject) × 2 (Sex, Between-subject) repeated-measure general linear model. Platform placement error, or spatial precision, was the outcome of interest, predicted by map cue type, age (continuous, sample mean-centered), and participant sex, as well as two- and three-way interactions between these variables in the regression. Prior experience with 3D video games and symptom questionnaire scores for motion sickness were included as covariates.

Data were analyzed using IBM SPSS Statistics, version 28.0.0.0. Data were examined before the analyses to ensure that assumptions of multivariate regression were met. Eleven cases were identified as multivariate outliers (χ^2^ = 26.125, α = 0.001, d*f* = 8). The models were evaluated with and without outlier cases and revealed no substantive differences that could have biased the interpretation. Therefore, all findings are reported for the complete sample. All other assumptions of the multivariate linear model with repeated measures except for sphericity (χ^2^ = 190.04, d*f* = 5, *p* < 0.001) were met. The effect of this violation was controlled by using, Pillai's *F*-statistic, a robust estimate. Significance for all hypothesis testing was set at α = 0.05, and planned least significant difference (LSD) pairwise comparisons were used in *post-hoc* simple effects analyses to decompose omnibus effects. Based on the results of the hypothesis tests, an exploratory multiple regression model was estimated to test map recall placement error in the all-cue condition, using the same predictors with the addition of a free map recall score. Significance was adjusted to α = 0.01 to control for type I error in the exploratory analysis.

## 3 Results

### 3.1. Navigation efficiency during learning trials

Pearson correlations among map recall measures and average distance traveled (log-transformed) in the vMWM are reported in Table 2. They indicate a moderate to strong association between navigation efficiency and map recall. A negative correlation was found between the average distance traveled and free recall of the map (*r* = −0.348, *p* < 0.001), indicating that less efficient navigation was associated with fewer correctly recalled environmental details, in accordance with the impoverished cognitive map hypothesis. This was further supported by the results of the cued map recall: less efficient navigation correlated with greater platform placement error when only proximal cues were available (*r* = 0.493, *p* < 0.001) and for all cues combined (*r* = 0.453, *p* < 0.001), with a similar but non-significant trend for only distal cues (*r* = 0.124, *p* = 0.11).

### 3.2. Differences in spatial precision at recall by cue type

A 3 (Cue Type) × 2 (Sex) repeated-measure general linear model revealed that platform placement error differed with the type of cue available at subsequent recall (Cue Type, *F*_2, 162_ = 4.32, *p* = 0.02, $\eta_{p}^{2}$ = 0.05). The greatest placement error (or worse spatial precision) occurred with distal cues (*M* = 16.21; SD = 10.55) as compared to proximal ones (*M* = 9.78; SD = 5.94, *t*_168_ = 7.49, *p* < 0.001) or combined cues (*M* = 9.98; SD = 6.10, *t*_168_ = 7.51, *p* < 0.001), regardless of age and sex. There was no difference in placement error when comparing recall with proximal cues to combined cues (*t*_168_ = −0.53, *p* = 0.30). Neither of the control variables—the 3D game experience (*F*_2, 162_ = 1.11, *p* = 0.33) and the motion sickness symptom profile (*F*_2, 162_ = 0.26, *p* = 0.77)—was related to platform placement error in the model.

#### 3.2.1. Age- and sex-related differences in spatial precision at recall

**Figure 2:**
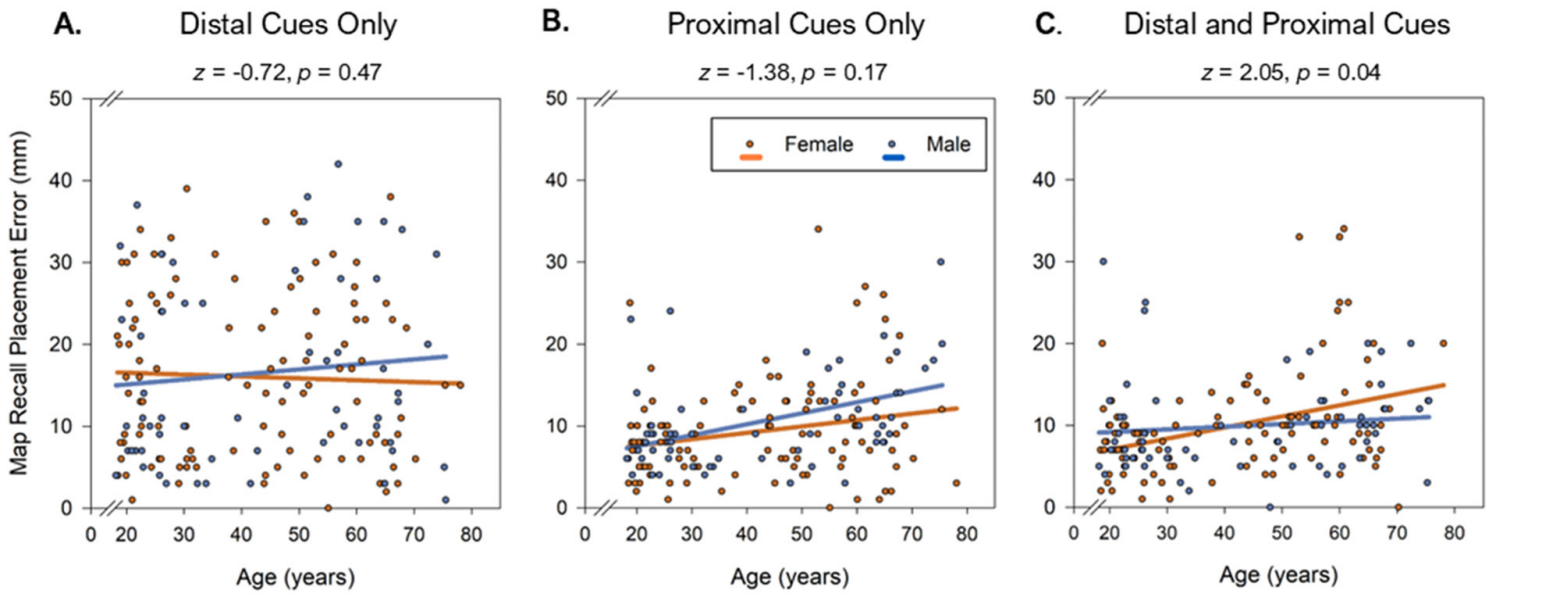
**(A–C)** A general age-related deficit in spatial precision is evident when provided only distal landmark cues, only proximal cues, or when provided all cues combined. Sex significantly moderated this effect only during recall with all cues combined; greater placement error with advanced age was observed in middle-aged and older women, as compared to men, with the slopes crossing at approximately age 43 years.

Older age was associated with greater placement error across all cue types (Age, *F*_1, 163_ = 4.83, *p* = 0.03, $\eta_{p}^{2}$ = 0.03). This age effect did not, however, differ between the presentation of distal, proximal, or combined cues (Cue Type × Age, *F*_2, 162_ = 2.72, *p* = 0.07). There was no main effect of sex (*F*_1, 163_ = 0.05, *p* = 0.82, $\eta_{p}^{2}$ = 0.00). However, a significant two-way interaction of Cue Type x Sex (*F*_2, 162_ = 4.78, *p* = 0.01, η_*p*_^2^ = 0.06), as well as a three-way interaction of Cue Type × Age × Sex (*F*_2, 162_ = 8.50, *p* < 0.001, η_*p*_^2^ = 0.10) was observed. To determine the source of this effect, semi-partial correlations of age with placement error were compared between men and women with Fisher *z*-tests for each map cue type (see Table 3 and Figure 2). This *post-hoc* analysis revealed that the correlation between age and placement error among women was stronger than among men, only when combined cues were provided (*z* = 2.05, *p* = 0.04; Figure 2C).

**Table 3:** Semi-partial correlations among study variables stratified by sex.

	**Males (*****n =*** **61)**				**Females (*****n =*** **108)**			
**Variable**	**1**	**2**	**3**	**4**	**1**	**2**	**3**	**4**
1. Age	–				-			
2. Map 2: *Distal Cues*	0.05	–			−0.07	–		
3. Map 3: *Proximal Cues*			0.40^**^	0.12			–		0.20^*^	0.17	–	
4. Map 4: *All Cues*	0.02	0.25^*^			0.61^**^	–		0.34^**^				0.21^*^	0.68^**^	–

Semi-partial correlations of age with map recall placement error for men and women, controlling for 3D game experience and motion sickness symptom profiles.

^*^p < 0.05, ^**^p < 0.01.

### 3.3 Landmark-place association accuracy predicts spatial precision when all cues are provided

**Figure 3:**
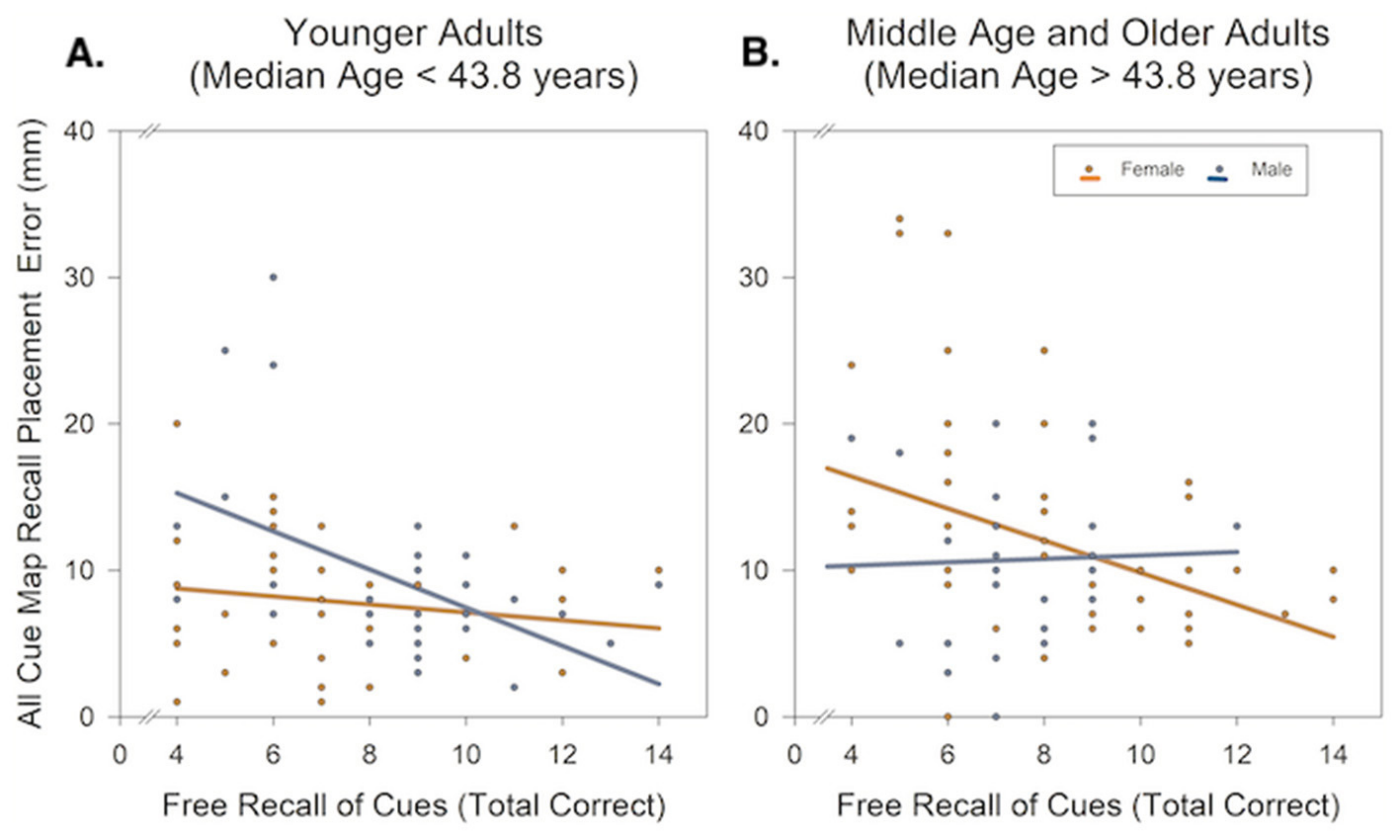
Age-by-sex related differences in the number of freely recalled virtual environment details predicting spatial precision when provided all cues together. The relation between free recall of cues and spatial precision differs between men and women by age. **(A)** Among young adults (Median age = 25.02 years), more freely recalled details was associated with greater spatial precision, and this effect was more prominent in young men as compared to young women. **(B)** Among middle-aged and older adults (Median age = 78.08 years), a similar effect was observed but was prominent among women and not men.

Better free recall of landmark-place associations was correlated with higher spatial precision when combined distal and proximal cues were provided (*r* = 0.28, *p* < 0.001; Table 2). Therefore, the Cue Type × Age × Sex interaction effect, which suggested greater age-related differences in spatial precision among women in a combined cues condition, may reflect recalling fewer details of the environment. An exploratory multiple regression model tested age, sex, free recall score, and their interactions predicting placement error when provided combined distal and proximal cues (adjusted α = 0.01). A significant three-way interaction indicated a complex association dependent on age and sex: Age × Sex × Free Recall, *F*_1, 159_ = 7.91, *p* < 0.01, $\eta_{p}^{2}$ = 0.05 (see Figure 3). *Post-hoc* comparison of simple slopes revealed a correlation between lower free recall of landmark-place associations and worse spatial precision. This effect was largely driven by middle-aged and older women, particularly those aged 43.8 years and older (see Table 4). Notably, this association was attenuated for younger women and middle-aged and older men. This suggests that even when recalling fewer details of the environment, these participants could use landmarks to improve precision. In young adults, a significant negative correlation was observed among men. Visual evaluation of the scatterplot suggests that this correlation could be leveraged by a few individuals with very large placement errors. Taken together, this suggests that the availability of distal and proximal cues together may not be universally beneficial to older adults' spatial precision, particularly for older women who recalled fewer landmark details.

**Table 4:** Simple slopes analysis of age-by-sex differences in recall placement error.

**Age group**	**Age**	**Sex**	**Effect**	***p***	**LLCI**	**ULCI**
Younger adults	22.25	Female	−0.07	0.83	−0.72	0.58
	22.25	Male	−1.26	<0.01	−2.08	−0.43
Middle-aged adults	43.83	Female	−0.71	<0.01	−1.15	−0.28
	43.83	Male	−0.65	0.05	−1.3	0.01
Older adults	64.02	Female	−1.31	<0.01	−2.10	−0.52
	64.02	Male	−0.07	0.88	−1.06	0.91

The simple slopes analysis suggests that age 43.8 years was a point in the continuous age scale where the relation between free recall and all cue placement error deviated.

LLCI, lower limit 95% confidence interval; ULCI, upper limit 95% confidence interval.

## 4 Discussion

Deficits in wayfinding ability contribute to the loss of independence among older adults and augur the onset of dementia. Identifying age-related differences in the amount and precision of recalled environmental detail can provide insight into the sources of decline in wayfinding ability. In a cross-sectional sample of cognitively unimpaired and reasonably healthy adults, we found that after completing a vMWM task, participants' landmark recall depended on the type of navigation cues available. Presentation of proximal cues, alone or in combination with distal cues, was associated with greater spatial recall precision. There was an unexpected sex difference in the magnitude of this effect: middle-aged and older women committed more substantial placement errors with increasing age than men of any age did. An exploratory analysis further suggested that this effect was primarily driven by middle-aged and older women who recalled fewer landmark-place associations and subsequently had worse spatial precision in marking the location of the platform when provided all cues. The findings discussed here highlight relevant individual differences in cue usage and landmark recall by older adults, with implications for cognitive map development and subsequent wayfinding behaviors.

Consistent with a general age-related deficit in the generation and use of a cognitive map as suggested in the literature (Wilkniss et al., 1997; Moffat and Resnick, 2002; Iaria et al., 2009; Montefinese et al., 2015; Muffato et al., 2021), advanced age correlated with worse spatial recall precision. A deficit in the generation, maintenance, and use of a cognitive map is presumed to underlie the commonly reported age-related deficits in navigation accuracy and efficiency during task performance (Moffat and Resnick, 2002; Rodgers et al., 2012; Daugherty and Raz, 2017). A substantial amount of evidence suggests that cognitive processes essential to the formation and use of a cognitive map decline with age (Iaria et al., 2009). Episodic memory is crucial for recalling experienced events, including their spatial and temporal details (Nadel and Payne, 2002; Fan et al., 2023), and is a key correlate of the cognitive map (O'Keefe and Nadel, 1978). Episodic memory ability partially depends on hippocampal circuits, including its neural computational processing that facilitates binding pieces of information to one another, to location and in time based on experience (Ryan et al., 2010; Moscovitch et al., 2016). The discovery of place cells in the hippocampus and grid cells in the adjacent entorhinal cortex have instantiated these regions as key neural substrates for knowledge of the environment, its landmark cues, goal locations and the routes traveled to get there, all represented as a coherent representation to be referenced for future travel (Eichenbaum, 2017). With advancing age, the hippocampus experiences accelerated shrinkage and functional decline, contributing to well-documented deficits in wayfinding, including the creation and use of a cognitive map. Age-related shrinkage in the hippocampus correlates with worse spatial encoding and recall of environmental details (Moffat et al., 2007; Head and Isom, 2010), and less efficient wayfinding (Daugherty et al., 2016; Daugherty and Raz, 2017). Additionally, reduced connectivity between the hippocampus and cortical regions such as the prefrontal cortex may impair the flexible use of stored spatial information during goal-directed navigation (Dahmani and Bohbot, 2015; Lester et al., 2017). Therefore, age-related declines in neural correlates like the hippocampus, in combination with deficits in spatial processing and working memory, likely contributes to an incomplete or inaccurate cognitive map in older adults (Hertzog and Rypma, 1991; Begega et al., 2001; Iaria et al., 2009; Merhav and Wolbers, 2019; Zhou et al., 2022). Although we did not examine neural correlates in the present report, the pattern of evidence we found here can inform future longitudinal studies, which are necessary to estimate age-related changes in brain structure and wayfinding correlates accurately.

Age-related differences in the accurate use of a cognitive map are viewed as a reflection of a greater reliance on proximal cues over distal ones (Moffat and Resnick, 2002; Rodgers et al., 2012; Kimura et al., 2019; McAvan et al., 2021). However, this finding was not replicated in the current study. First, providing only distal cues at subsequent recall elicited less precise spatial recall, regardless of age. This general effect may stem from an insufficient number of distal landmarks provided in this experiment (Rodgers et al., 2012), or the use of wall features in the virtual room may have been less salient for everyone. Second, contrary to the extant reports (Moffat and Resnick, 2002; Rodgers et al., 2012; Kimura et al., 2019; McAvan et al., 2021), advanced age was associated with reduced spatial precision, even when only proximal cues were provided. While older adults may indicate a preference for proximal cues, they may find it difficult to effectively use this information due to age-related declines in associative memory binding (Naveh-Benjamin, 2000), or because of a reduced ability to encode contextual information (Spencer and Raz, 1995), and these deficits may be amplified when placed in unfamiliar or complex environments (Naveh-Benjamin, 2000; Zhong and Moffat, 2016). Importantly, in this study, the age-related differences in spatial recall precision were relatively similar across cue types, suggesting a general recall inaccuracy rather than a shift in cue preference or utilization.

Discrepancy with prior reports may be due to differences in the number of navigation trials used in the studies, during which landmark-place associations are learned. In this study, map recall tasks were administered after 25 learning trials, which is sufficient for older adults to reach stable navigation performance levels even with slowed acquisition (Daugherty et al., 2016). The other reports used fewer learning trials; therefore, age-related differences in spatial precision, varying by cue type, might have reflected differences in navigation learning rather than subsequent recall. While this study did not directly examine search behaviors during completion of the vMWM paradigm, it is plausible that observed age-related differences could be due in part to the quality of spatial search strategies used. A recent study revealed poorer quality of exploration behaviors in middle-aged adults as compared to their younger counterparts, and a concomitant reduction in wayfinding success (Puthusseryppady et al., 2024). Another study, with a vMWM design similar to this study but with only 10 learning trials, found that allocentric strategy preference benefited vMWM performance for young adults, and had no effect in older adults (Rodgers et al., 2012). It is plausible that inefficient search (i.e., spending less time exploring the goal quadrant) and slower encoding would influence subsequent recall of pertinent environmental details, thus affecting the overall quality of the cognitive map with limited learning trials. Our results suggest that when given sufficient exposure, age-related differences in spatial recall precision are independent of the type of navigation cue available. Evidence of age differences in spatial recall precision even after many repeated learning trials resembles real-world navigation in familiar environments, in which severe wayfinding deficits would be a source of concern for dementia risk.

When considering the potential influence of sex, a curious pattern of age-related differences emerged: worse spatial precision was observed in middle-aged and older women compared to the rest of the sample, but only when both proximal and distal cues were presented at recall. While the effect appears to be driven by older women performing notably worse, and a few younger men whose poor performance may reflect a lack of motivation rather than a memory deficit, the pattern of results persisted even when these leverage cases were removed as outliers. This is consistent with noted sex differences in navigation, with men as superior navigators (Astur et al., 1998; Iaria et al., 2009; Chai and Jacobs, 2010; Liu et al., 2011; Munion et al., 2019). Our findings suggest a more nuanced view, given the absence of a main effect of sex in this sample and is in line with several reports of age-by-sex interactions driven by older women performing worse than the rest of the sample in visual attention of allocentric landmarks and route knowledge (Wang et al., 2018) and wayfinding efficiency (Rodgers et al., 2012). This direction of interaction is not consistently replicated across various tasks (Yu et al., 2020).

In further explorations of this unexpected effect, researchers may consider that more landmark cues available presumably leads to more accurate recall of the platform location (Bates and Wolbers, 2014; Merhav and Wolbers, 2019). To understand the source of this effect, free recall of landmark-place associations (an index of the detail in the individual's presumed cognitive map) was tested as a predictor of spatial recall precision of the platform location. On average, individuals who freely recalled more accurate details were also more accurate in placing the platform, in accord with other reports (Reggente et al., 2020). This is consistent with the general understanding of the cognitive map storing relevant details of objects and location that can be used to recall goal locations and plan routes (Tolman, 1948; Weisberg and Newcombe, 2016; Epstein et al., 2017). Somewhat paradoxical, our findings revealed that middle-aged and older women who freely recalled fewer landmark-place associations had *worse* spatial precision for marking the goal platform when provided a complete picture of the environment. Notably, this association was attenuated in young women and middle-aged and older men, which suggests that even when recalling fewer details, they could use those landmarks for good spatial precision of the goal location. A study of eye tracking during a wayfinding task revealed that women had longer periods of fixation, which has been linked to an increase in memory encoding demands, as compared to men who demonstrated greater exploration of the arena in a virtual maze environment (Mueller et al., 2008). In a real-world indoor experiment involving navigation of a mall, participants were provided with a map and route memorization instructions before navigation, while wearing mobile eye-tracking glasses (Wang et al., 2018). In this study, older women, in particular, had the longest fixation time and map reading durations; they fixated longer on store allocentric cues than doorways and elevators during navigation; and made more navigation errors with worse route recall (Wang et al., 2018). A qualitative analysis of participants' descriptions of their preferences and experiences suggested that women had a stronger preference for allocentric landmark-based navigation, especially at decision junctures. However, with age, there was a general shift toward an egocentric preference (Wang et al., 2018). This set of findings may describe the unique circumstance of an older woman who may prioritize more detailed, context-rich encoding strategies that are more difficult to sustain in aging (Spencer and Raz, 1995) and are more error-prone due to unremembered landmarks.

When taken together, these findings suggest that older women are vulnerable to cognitive interference (Davis et al., 2008; Muecke et al., 2018) and their cognitive maps are more susceptible to irrelevant or distracting information. In other words, they may suffer from lower signal-to-noise ratio in comparison to men or younger women. Although all cues were designed to be equally relevant for navigation in the task, deployment of all of them at once is not necessary for accurate navigation. Across learning trials, participants could identify and encode landmark-place relations based on their experience and strategy preference. Recent studies comparing landmark-goal distances on performance in a virtual navigation task revealed that cues in proximity to the platform overshadowed the learning of more distal cues (Herrera et al., 2022, 2023). Considering our findings, it is plausible that older women may be unable to adequately disregard information that does not align with their cognitive map—a situation where having more information available at the time of recall can lead to wayfinding errors. Such interference is reminiscent of other instances in which older adults found seemingly helpful, context-rich environments and stimuli to be challenging (Bender et al., 2017). This highlights how a potential overabundance of cues may interfere with effective wayfinding and underscores the need for more intentional design in built environments. For example, distinct architectural features and large building labels that can be viewed from afar at decision junctures would align with both allocentric and geometric preferences and encourage accurate wayfinding for older adults (Wiener and Pazzaglia, 2021).

Additional sources of individual differences are also worth considering. Importantly, various societal and experiential factors—including disparities in access to navigational experiences, or differential use of transportation systems based on geographical characteristics—may shape differences in wayfinding behavior across the lifespan (Spiers et al., 2023). Moreover, given the complex age-by-sex effect, menopause and related factors may contribute to vulnerability of wayfinding after middle age (Chapin et al., 2020). The drop in estrogen that is associated with the onset of menopause can have a systemic impact on the body, resulting in increased inflammatory markers, reduced mitochondrial activity, and more (Conde et al., 2021). Women experiencing menopause-related changes are at greater risk for cognitive decline (Reuben et al., 2021; Sochocka et al., 2023), which could lead to impaired spatial learning and wayfinding ability. This may be partly due to accelerated volumetric decline of key brain regions—including the hippocampus and medial temporal lobe and frontal cortices, and the striatum in women experiencing menopause (Ramli et al., 2023). Moreover, when considering hormone-specific changes, neuroimaging evidence has suggested that the initiation of hormone replacement therapy closer to the onset of menopause can reduce the rate of cortical but not necessarily hippocampal shrinkage (Raz et al., 2004) as compared to delayed initiation or no implementation of treatment, though this doesn't always translate to improvements in spatial learning and memory (Erickson et al., 2010), highlighting the complexity of the relationship between hormones, the brain, and cognitive performance. These hormone-related changes likely interact with broader physiological changes that co-occur with advanced age and menopause, including physical frailty and related declines in mobility that are associated with age-related cognitive decline (Demnitz et al., 2016; Borges et al., 2019), and may have a synergistic effect on wayfinding. While these are currently only speculations based on the reported results, they underscore the need for continued investigation to understand better age - and sex-related differences in the creation of cognitive maps and their use for wayfinding.

### 4.1 Limitations

The results of this study should be viewed in light of its strengths and limitations. The findings reported here were obtained in a cross-sectional study, which precludes testing age-related change and individual differences therein (Raz and Lindenberger, 2011; Fjell et al., 2013). It is important to note that our sample of convenience was unbalanced with respect to sex, with women outnumbering men. Such lack of balance may limit the generalizability and introduce biases when interpreting the observed sex differences. In addition, we did not examine the potential cognitive and neural correlates of age and sex differences in navigation that are described across the literature. Such analyses will be presented soon, based on data being collected in our lab. Although the vMWM task is a well-validated laboratory assessment that correlates with real-world navigation (Astur et al., 1998; Hamilton et al., 2002), it has weak ecological validity in terms of everyday human experiences. In addition, the map reproduction tasks were drawn from an overhead perspective, whereas participants completed the vMWM from a confrontational first-person view. This discrepancy could have introduced additional cognitive demands for mental rotation that are also vulnerable in aging. Restructuring the maze environment using immersive virtual reality technologies to evaluate navigation behaviors may help to overcome these limitations. Nonetheless, the laboratory control of contextual factors in the virtual task and the minimization of the confound of physical motor deficit provide an externally valid and robust measure of cognitive processes supporting navigation.

### 4.2 Conclusion

We observed that older age was associated with worse precision in subsequent spatial recall, independent of cue type, indicating non-specific deficits in the cognitive processes essential for cognitive map development and use. Furthermore, we observed that age differences in spatial recall precision varied between men and women. A stronger correlation was found between age and placement error for women, but only for combined proximal and distal cues. This specific effect was partially explained by free recall of landmark-place associations, as middle-aged and older women who explicitly recalled fewer cues subsequently had worse spatial recall precision when provided a complete picture of the environment. Taken together, consideration of age- and sex-related deficits in landmark details included in a cognitive map can provide insight into declines in wayfinding abilities in real-world navigation, and how these deficits may be used to identify individuals at risk for dementia.

## Data Availability

The raw data supporting the conclusions of this article will be made available by the authors, without undue reservation.
